# Improving Human Health in China Through Alternative Energy

**DOI:** 10.3389/fpubh.2021.613517

**Published:** 2021-04-21

**Authors:** Melissa Scott, Robert Sander, Gregory Nemet, Jonathan Patz

**Affiliations:** ^1^Duke Samuel DuBois Cook Center on Social Equity, Duke University, Durham, NC, United States; ^2^Air Quality and Greenhouse Gases Program, International Institute for Applied Systems Analysis (IIASA), Laxenburg, Austria; ^3^La Follette School of Public Affairs, University of Wisconsin-Madison, Madison, WI, United States; ^4^Global Health Institute, University of Wisconsin-Madison, Madison, WI, United States

**Keywords:** air pollution, coal-fired power plants, renewable energy, China, human health

## Abstract

In this study, we estimate the health benefits of more stringent alternative energy goals and the costs of reducing coal-fired power plant pollution in China projected in 2030. One of our two overarching alternative energy goals was to estimate the health benefits of complete elimination of coal energy, supplemented by natural gas and renewables. The second was a policy scenario similar to the U.S. 2013 Climate Action Plan (CAP), which played a pivotal role leading up to the 2015 Paris Climate Agreement. We used the Greenhouse Gas and Air Pollution Interactions and Synergies (GAINS) model created by the International Institute for Applied Systems Analysis for our model simulations. We found that 17,137–24,220 premature deaths can be avoided if coal energy is completely replaced by alternative energy, and 8,693–9,281 premature deaths can be avoided if coal energy is replaced by alternatives in a CAP-like scenario. A CAP-like scenario using emission-controls in coal plants costs $11–18 per person. Reducing coal energy in China under a CAP-like scenario would free up $9.4 billion in the annual energy budget to spend on alternatives, whereas eliminating the cost of coal energy frees up $32 billion. This study's estimates show that more stringent alternative energy targets in China are worth the investment in terms of health.

## Introduction

Climate change affects the health of populations across the globe as temperatures and sea levels rise, and weather becomes more extreme (1, 2). Because energy production remains the primary driver of greenhouse gas emissions (2), addressing the energy-use roots of global climate change ultimately affects human health. The most carbon-intensive energy source is coal. Burning coal emits about twice as much carbon dioxide (CO_2_) per unit of energy compared to the combustion of natural gas (3). In addition, burning coal generates emissions of fine particulate matter (PM_2.5_) or particles measuring <2.5 micrometers. PM_2.5_ travels deep into human airways causing cell damage to the lungs, which can lead to inflammation, cytotoxicity, cell death, as well as increases in cardiovascular disease, respiratory disease, lung cancer, asthma, and death (4, 5). PM_2.5_ also impacts brain functioning and mental health (6). A considerable number of published scientific literature shows a causal relationship between mortality and exposure to PM_2.5_ (5).

Therefore, reducing coal-fired power plant emissions to mitigate climate change can simultaneously decrease hazardous PM_2.5_. This represents a side benefit, or health “co-benefit,” of climate change policy measures (7). However, society is still reliant on fossil fuel energy. Three thousand two hundred and seventy-two coal-fired power plants exist worldwide with 1,199 more proposed (8). Of these 1,199 new coal-fired power plants, over 1/3rd of the capacity will come from China (8). Moreover, China accounts for 46% of coal consumption worldwide, followed by the United States (13%) and India (9%) (8). China also accounts for 20% of the global population (9) and has experienced a recent rise in its air pollution index (10). Thus, the large and rapidly growing Chinese population is exposed to an increasingly large amount of coal pollution with 13% of Chinese cities severely polluted, particularly during winter months when many places in China rely on coal as the main source for heating (10).

In this study, we estimate the health benefits and costs of reducing coal-fired power plant pollution to see whether more stringent alternative energy goals are worth the investment in China. This is important because it may not take much investment (or less investment than originally thought by policymakers) to reduce coal usage more aggressively in China in the coming years. For example, supplementing coal energy usage with alternatives and better controlling coal in the U.S. under the 2013 Climate Action Plan (CAP) would have prevented up to 6,000 annual deaths, 3,000 heart attacks, and 150,000 asthma attacks annually in the United States (11). The U.S. Climate Action Plan was issued in 2013 under President Obama and played a pivotal role in the international Paris Agreement on Climate Change in 2015, even though President Trump signed an executive order to rescind it in March, 2017. It was pivotal, in part, because the U.S. has never signed on to any international environmental agreement (such as the Paris Climate Agreement or the Montreal Protocol on Substances that Deplete the Ozone Layer), without first having domestic regulation on the pollutant or environmental hazard. CAP would have been the U.S.'s first domestic regulation on climate change, and was a signal to the rest of the world, particularly China and India, of the U.S.'s serious energy concerns and intentions.

In order to examine whether a more aggressive alternative energy transition currently planned in China might be worth the investment, we developed two overarching alternative energy goals: one scenario inspired by CAP wherein coal-fired power plant pollution is reduced by 32%, and elimination of coal energy and its pollution entirely. Elimination of coal energy involves completely replacing this energy source with alternative energy. Alternative energy consists of natural gas and renewables (solar thermal, solar photovoltaic, wind, nuclear, and geothermal). A CAP-like policy replaces coal energy with alternatives in China by about a third in the year 2030. In the U.S., CAP would have given states the flexibility to switch to alternative energy and/or increase existing pollution controls in modern coal plants by 2030, as long as a 32% reduction of coal pollution was achieved, and forced states to phase out the oldest and dirties plants (11, 12). Thus, we created variations of the CAP-like policy wherein alternative energy replaces coal and pollution controls are increased in modern coal plants to achieve a 32% reduction.

## Materials and Methods

Our study uses the Greenhouse Gas and Air Pollution Interactions and Synergies (GAINS) model created by the International Institute for Applied Systems Analysis (IIASA) to estimate the health benefits and emission-control costs of our energy scenario goals across all 32 regions of China. GAINS estimates the dispersion of pollutants in the atmosphere from energy sources, the concentrations of such pollutants as they mix with other pollutants in the atmosphere, the impacts of these pollutants on human health, and the future costs of emission-control technologies within energy sources (13). The five energy scenarios we developed are as follows:

**Scenario 1**: 100% reduction of coal-fired power plant emissions wherein coal energy is completely replaced by renewable energy;
**Scenario 2**: 100% reduction of coal-fired power plant emissions wherein coal energy is completely replaced by natural gas;
**Scenario 3**: 32% reduction of coal-fired power plant emissions wherein 32% of coal energy is replaced by renewable energy
**Scenario 4**: 32% reduction of coal-fired power plant emissions wherein 32% of coal energy is replaced by natural gas
**Scenario 5**: 32% reduction of coal-fired power plant emissions using emission-control technologies within coal plants

To calculate the health benefits and emission-control costs of our alternative energy targets we change the petajoules (PJ) of energy produced by individual energy plants across all 32 regions in China within the model. The first step in changing the PJ of energy produced by renewable and natural gas plants is to calculate the kiloton change of PM_2.5_ pollution from coal plants. Knowing the kiloton change of PM_2.5_ from coal plants allows us to calculate the amount of coal required across China. Once the kiloton change of PM_2.5_ is calculated, the second step is to input each plant's decreased coal-derived PJ value. Step three is to compare the PJ values of the renewable or natural gas energy needed to replace the decreased coal power. When coal energy is replaced and primary PM_2.5_ is reduced, the GAINS model reduces NO_X_ and SO_2_, as well as the greenhouse gases (GHG) CO_2_ and CH_4_ in order to correspond with the changed energy supply.

A detailed explanation of these steps are provided in the GAINS User Guide (available upon registering at the Model Interface) (14), which describes how to create a scenario using the model from multiple pollutant sources. This guide explains how a user can download the energy activity data, manipulate the activities, and upload the new activity data through the GAINS interface. The basic math for the model is: Emissions = Activities ^*^ Emission Factors. The main interaction with the model is done by the Excel Files upon upload.

GAINS uses the Unified European Monitoring and Evaluation Programme (EMEP), a Eulerian model, to calculate emission changes in the atmosphere involving more than 100 chemical reactions of 70 chemical pollutants produced (15, 16). After the outcomes are ascertained by EMEP, GAINS then estimates the premature mortality due to a pollutant change compared to baseline.

GAINS uses findings from the World Health Organization (WHO) review on the health impacts of air pollution (17, 18) and the results of the American Cancer Society cohort study (19) and its subsequent analysis (20) to quantify premature mortality attributable to long-term PM_2.5_ exposure (13).The relative risk function from Pope et al. is based on premature mortality due to cardiopulmonary disease and lung cancer from PM pollution in cohorts 30 years old and above. GAINS uses cohort- and country-specific mortality data obtained from life table statistics (13). For all cohorts in a country *l* the change in life years *L*_*l*_ is then calculated in GAINS as the sum of the change in life years for the cohorts living in the grid cells *j* of country *l*:

(1) $$\begin{array}{l} \Delta L_{l}=\;\sum_{c=W_{0}}^{W_{l}}\Delta L_{c,i\;}=\;\beta \sum_{j\in l}PM_{j}\frac{Pop_{j}}{Pop_{l}}\;\sum_{c=w_{0}}^{W_{l}}Pop_{c,l\;}\\ \;\int_{c}^{W_{l}}l_{c}\left(t\right)\log l_{c}\left(t\right)dt \end{array}$$

where

→ *Delta L*_*c,l*_ – Change in life years lived for cohort *c* in country *l*.
→ *Pop*_*c,l*_– Population in cohort *c* in country *l*.
→ *Pop*_*j*_– Total population in grid cell *j* (at least of age *w*_*o*_ = 30)
→ *Pop*_*l*_– Total population in country *l* (at least of age *w*_*o*_ = 30)

The GAINS energy activity data comes from the International Energy Agency statistical energy and process data, specifically the World Energy Outlook 2020 report (21), for projections until 2030. Every 5 years, China submits a new legislative plan. GAINS draws on the 13th Five Year Plan legislation, drafted between 2016 and 2020 for its Baseline Current Legislation scenario dataset. The baseline dataset is roughly compatible with CO_2_ emissions for a 6°C warming by 2,100.

### Costs

The first step in the cost estimate is to calculate how much pollution each coal plant's emission-control technology is able to remove in 2030. Then we alter the percentage at which each emission-control is “on” to achieve the same pollutant reduction as the alternative energy CAP-like targets. Since it is impossible to control emissions from coal plants using control technologies by the same amount as switching to 100% alternative energy (Scenarios 1 and 2), we were only able to measure the emission control costs of a CAP-like target.

Coal power plant emission-control technologies include dedusters, electrostatic precipitators and fabric filters. Their costs are technology based and account for the structural differences of plants, their fuel use patterns, the amount and quality of the coal burned, and the control measures already applied (13). To estimate the costs for applying a technology in a given country, GAINS considers international pricing data for technologies and adjusts them to country-specific conditions, taking into account local labor costs, energy prices, and costs of by-products (22). All costs are in Euro 2005 (14).

GAINS estimates the costs of each emission control technology considering annualized investments (I^an^), fixed (OM^fix^), and variable (OM^var^) operating costs, and how they depend on technology m, country i and activity type k (23–30). GAINS assumes technological progress in the performance and cost data, based on literature estimates (13). A Unit cost of abatement (ca) of coal-fired air pollution, related to one unit of activity (A), add up to:

(2) $$\begin{array}{r} {ca}_{i,k,m}\;=\;\frac{I_{i,k,m}^{an}+\;{OM}_{i,k,m}^{\mathrm{fix}}}{A_{i,\;k\;}}\;+\;{OM}_{i,k,m}^{var} \end{array}$$

## Results

### Health Benefits

**Figure 1:**
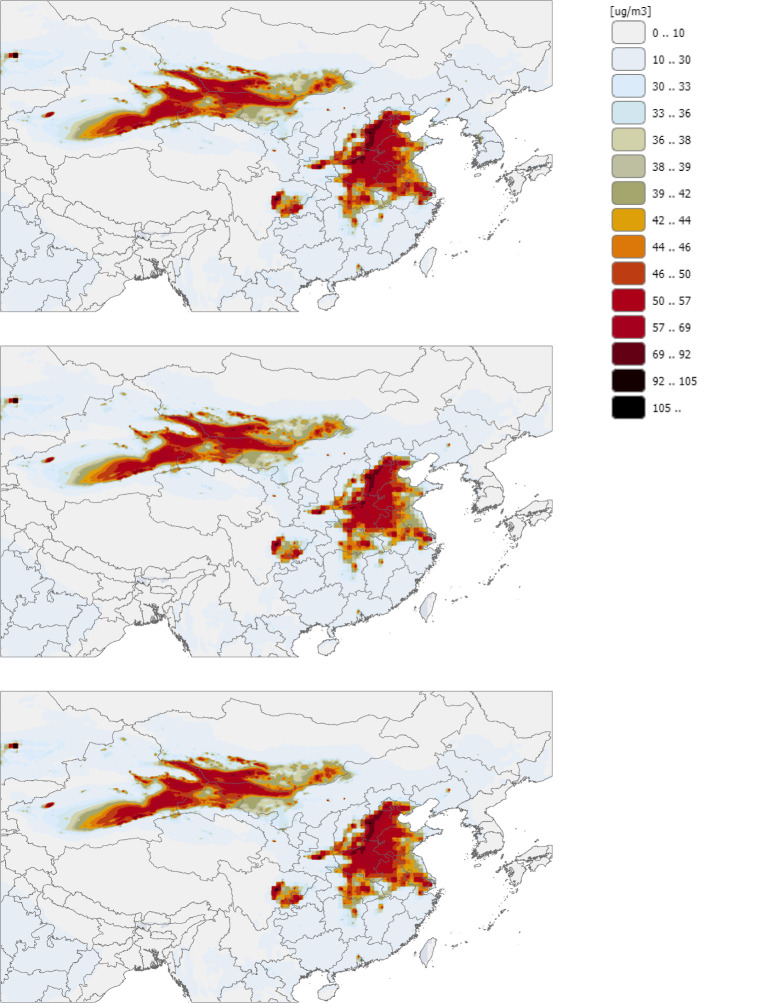
**Concentrations of PM**_**2.5**_ **projected in China in 2030 under three energy scenarios**. The top map shows the business-as-usual scenario (0% reduction of coal-fired power plant emissions). The middle map shows Scenario 1 wherein coal-fired power plant emissions are eliminated and renewable energy replaces coal. The bottom map shows Scenario 3 wherein coal-fired power plant emissions are reduced by 32% and renewable energy replaces coal.

Each of our five scenarios and their health results are found in Table 1. Under Scenario 1 wherein coal-fired power plant pollution is eliminated and replaced by renewables, 24,220 premature deaths are avoided. Under Scenario 3 wherein coal-fired power plant pollution is reduced by 32% and renewables replace coal to meet energy demand, 9,281 premature deaths are avoided. Under Scenario 5 wherein coal-fired power plant pollution is reduced by 32% using control technologies, 4,906 premature deaths are avoided. These are low estimates, since they exclude Chinese people <30, which is projected to be 37% of the population in China in 2030 (31), and younger populations such as infants and young children experience some of the worst health impacts from air pollution (32–36). The largest changes in coal-fired power plant air pollution are seen throughout Eastern China, as shown in Figure 1.

**Table 1:** Energy scenarios' reductions of PM_2.5_, NO_x_, and SO_2_, their corresponding health results compared to Business-as-usual, and greenhouse gas reductions.

	**Description**	**Premature deaths avoided**	**GHG reductions from coal**
Scenario 1	100% reduction, replaced by Renewables	24,220	100% reduction
Scenario 2	100% reduction, replaced by Natural Gas	17,137	100% reduction
Scenario 3	32% reduction, replaced by Renewables	9,281	32% reduction
Scenario 4	32% reduction, replaced by Natural Gas	8,693	32% reduction
Scenario 5	32% reduction using emission-control technologies in coal plants	4,906	0% reduction

### Pollutant and Greenhouse Gas Emissions

In Scenarios 1–4, reductions in PM_2.5_, NO_X_, and SO_2_ correspond with a reduction in GHGs. However, reducing PM_2.5_, NO_X_, and SO_2_ using control technologies alone does not reduce GHG emissions, as demonstrated in Scenario 5. This difference is noteworthy when factoring in the health co-benefits of mitigating climate change. It is important to note that in Scenario 4, where coal is replaced by natural gas, methane emissions are reduced by 32% at the coal plant, but methane leakage from hydraulic fracturing procedures is not considered because the GAINS model does not include the full life cycle of fossil fuels.

### Costs

As shown in Table 2, implementing more restrictive emission-controls within coal power plants for 365 days in 2030 saves 4,906 premature deaths in China under a CAP-like target and costs 13.5 billion Euros. In a population of 889 million people over the age of 30 in China, this translates to 15.85 Euros or $17.74 per person. In a total population of 1.4 billion people projected in 2030, this translates to 9.64 Euros or $11.33 per person.

**Table 2:** Energy scenarios' emissions reductions, premature deaths avoided, and costs compared to Business-as-usual (BAU).

**Scenario**	**BAU**	**1**	**2**	**3**	**4**	**5**
Coal-fired power plant emissions reductions in 2030	0%	100%, replaced by renewables	100%, replaced by natural gas	32%, replaced by renewables	32%, replaced by natural gas	32% using emission control technology
Premature deaths avoided	0	24,220	17,137	9,281	8,693	4,906
Emission control costs compared to BAU (Million Euros/Year)		−27,688	−27,688	−8,860	−8,860	13,541

While these costs are specific to emission-control technologies that control NO_X_, SO_2_, and PM_2.5_ and not to the price of alternative energy, when less coal is burned and less emission-control technologies are implemented. Under the alternative energy targets, wherein 8,693–9,281 premature deaths are avoided by replacing coal with renewables or natural gas under a CAP-like policy, eight million Euros are saved on operating costs. Similarly, when 17,137–24,220 premature deaths are saved by completely replacing coal with alternatives, 27 billion Euros are saved on what would have been spent on coal-fired power plant emission-control technologies and their upkeep. This frees up 8–27 billion Euros ($9.4–32 billion) in the 2030 annual energy budget to spend on alternative fuels.

### Uncertainties

There are two major sources of uncertainty using the GAINS model. The first is the use of EMEP, the Eulerian model. In using EMEP, the atmospheric dispersion and therefore PM_2.5_, NO_x_, and SO_2_ emissions may be underestimated in GAINS because the methods used only measure the changes in precursor emissions and do not include the rather unknown role of secondary organic aerosols and natural sources (13).

Second, the relative risk of particulate matter from Pope et al. is based on premature mortality due to cardiopulmonary disease and lung cancer from PM pollution. However, it does not include premature mortality due ischemic heart disease, stroke, cerebral vascular disease, and lower respiratory infections in children which other studies have used in their disease-specific mortality and morbidity estimates due to air pollution (37–48). While, cause-specific death rates may incur lower errors compared to all-cause mortality studies on air pollution (49), not including more of the disease-specific mortality for PM may lead to an underestimate in the premature deaths due to coal-fired power plant pollution.

## Discussion

The greatest health co-benefits in our study are evidenced in scenarios wherein coal energy is eliminated in 2030 and replaced by renewables. The greatest health benefits are in Eastern regions of China where coal is heavily used for energy and many people reside. From a policy perspective, it is important to take into consideration the values of people in regards to their health and physiological needs for cleaner air, as air pollution not only impacts cardiovascular health and respiratory health and even diabetes, but has been shown to impact mental health such as depression, anxiety and dementia as PM leads to oxidative stress to lower parts of the brain that effect brain functionality and mood.

Several studies have estimated the percentage of people in various regions of China willing to pay for cleaner air, as well as the amount they would be willing to contribute. Chu et al. found that 73% of people in Wuhan, China were willing to pay for air quality improvement (50). A study of 975 people in Shanghai found that 52% of the community and 70.2% of hospital populations were willing to pay between $68.50 and $80.70 to improve air quality, respectively (51). Sun et al. found that 90% of respondents in China are willing to pay for reducing air pollution, and that among those polled the average amount each individual was willing to pay was $56.18 (52). Lastly, Zhang et al. found that 78.5% of citizens in Nanchang, China expressed willingness to contribute money to improve air quality (53). This literature suggests that people have a relatively high willingness to pay for cleaner air in China. At $11–18 per person for a 32% reduction of air pollution that avoids 4,906 premature deaths, it seems reasonable from a policy perspective for China to transition to cleaner energy more aggressively.

There are major health costs with natural gas as an alternative to coal or as a bridge fuel to renewables that are not included in our health estimates. Some have referred to natural gas as a “bridge to nowhere” (54). For example, new evidence suggests hydraulic fracturing for natural gas impacts the birth weight of babies near fracking sites (55). Moreover, switching to natural gas reduces 588–7,083 less premature mortalities compared to renewables in Scenarios 1–4. However, natural gas consumption and production is projected to rise in China as it aggressively looks at ways to increase its volumes and has very ambitious shale gas targets (56, 57). China's technically recoverable shale gas reserves are almost 50% higher than those of the United States (58).

Natural gas production leaks methane which is 100-times more potent than CO_2_. Furthermore, standard bottom-up approaches to measure methane leakages can lead to gross underestimates (59, 60). Specifically, the U.S. Environmental Protection Agency's bottom-up approach of sampling methane emissions at selected utility company natural gas sites has estimated methane leakages at a 1.2% rate (61), whereas satellite data, tower samples, aircraft measurements, and other top-down studies have estimated leakages at much higher rates (62–64). For example, satellite data over Bakken and Eagle Ford formations in the U.S. have estimated methane leakages at a 6.2–10.2% rate. For there to be a net climate benefit of switching from coal-fired to gas-fired power plants, the methane leakage rate needs to be <3.2% (65). Thus, if leakages over 3.2% occur in China, natural gas is not a viable climate-friendly alternative energy.

It would be useful in a future analysis to obtain an estimate for the cost of renewable alternative energy. Estimates could be obtained by using International Energy Agency data and projections, interpreting China's 5-year legislation plans from 2016–2020, 2021–2025, and 2026–2030, and assessing future energy markets. Furthermore, it would be useful to measure the climate change health-related costs and benefits of our targets in a future analysis. For example, in 2030 the social cost of avoiding one ton of CO_2_ is estimated to be $50 (66). A 32% reduction of CO_2_ by switching to renewables avoids 2,079,250 Kt of CO_2_, which has a social cost of $104 million. West et al. found that the cost of cleaner energy worldwide is <$30/t CO_2_, whereas the benefit of cleaner energy is $200/t CO_2_, with two thirds of these benefits coming from China (67).

In conclusion, this study's estimates show that increasing alternative energy more aggressively than is currently planned in China is worth the investment in terms of health. Policymakers should consider getting off coal sooner and more aggressively, with considerable attention to a more rapid transition to renewables.

## Data Availability Statement

Publicly available datasets were analyzed in this study. This data can be found at: https://gains.iiasa.ac.at/gains/ASN/index.login?logout=1&amp;switch_version=v0.

## Author Contributions

MS performed data manipulation before input into the model and is the primary author of the text and tables. RS performed model data manipulation and output of Figure 1. GN and JP reviewed the paper. JP made contributions to the health results sections and health literature in introduction and discussion. GN gave input on the GAINS model, energy content, and framing of the health results for health and policy audiences. All authors contributed to the article and approved the submitted version.

## Conflict of Interest

The authors declare that the research was conducted in the absence of any commercial or financial relationships that could be construed as a potential conflict of interest.
